# Sodium restriction in patients with cirrhotic ascites: a protocol for a systematic review

**DOI:** 10.1186/s13643-016-0250-4

**Published:** 2016-05-10

**Authors:** Benjamin Walbaum, María Laura Valda, Gabriel Rada

**Affiliations:** Department of Public Health, Faculty of Medicine, Pontificia Universidad Católica de Chile, Marcoleta 434, Santiago, Chile; Emergency Department, Faculty of Medicine, Pontificia Universidad Católica de Chile, Lira 63, Santiago, Chile; Evidence-Based Health Care Program, Faculty of Medicine, Pontificia Universidad Católica de Chile, Santiago, Chile; Epistemonikos Foundation, Santiago, Chile

## Abstract

**Background:**

Avid renal sodium and water retention among other mechanisms produce ascites in patients with cirrhosis. The main guidelines recommend sodium intake reduction in order to counteract this complication. However, some randomized controlled trials have suggested a lack of benefit with a sodium-restricted over an unrestricted diet, and even an increase in ascites and renal complications has been reported. There are no systematic reviews addressing this question.

**Methods:**

A systematic review protocol has been designed and will be reported in line with Preferred Reporting Items for Systematic Reviews and Meta-Analyses Protocols (PRISMA-P). We will search for randomized controlled trials evaluating a salt-restricted versus unrestricted regime in patients with cirrhosis and ascites in EMBASE, MEDLINE, and the Cochrane Central Register of Controlled Trials. We will also try to identify literature by reviewing reference list of included studies and relevant reviews, screening main conference proceedings, and searching for unpublished and ongoing trials in the World Health Organization (WHO) International Clinical Trials Registry Platform.

Two researchers will independently undertake selection of studies, data extraction, and assessment of the quality of included studies.

We will estimate pooled risk ratios for dichotomous data and the mean difference or standardized mean difference for continuous outcomes. A random effect model will be used for meta-analyses. Data synthesis and other analyses will be conducted using RevMan software. Ethics and dissemination: no ethics approval is considered necessary. Results of this study will be disseminated via peer-reviewed publications and social networks

**Discussion:**

Sodium restriction is a widely accepted coadjuvant therapy for ascites; however, this indication is based primarily on expert recommendations. As far as we know, this will be the first systematic review assessing the effects of a sodium-restricted diet for ascites in cirrhotic patients.

Our systematic review will aim to provide a high-quality synthesis of current evidence for patients and clinicians about this question.

The main limitation might result from the reduced number and quality of primary studies available.

**Systematic review registration:**

PROSPERO CRD42015022161

**Electronic supplementary material:**

The online version of this article (doi:10.1186/s13643-016-0250-4) contains supplementary material, which is available to authorized users.

## Background

Hepatic cirrhosis represents the final stage of progressive and irreversible liver fibrosis observed in chronic hepatic disease. Liver architecture distortion determines portal hypertension. Added neurohumoral changes produce peripheral vasodilation, increased circulating volume, and sinusoidal vasoconstriction. The exact mechanism of peripheral vasodilatation is still unclear, but apparently, it is due to an imbalance between vasoconstrictors and vasodilators released as response to increased portal vascular resistance [1]. Circulating volume is increased primarily because of avid renal sodium and water retention due to relative hypovolemia sensed by the juxtaglomerular apparatus. Sodium and water reabsorption are mediated by increased secretion of renin, angiotensin, and aldosterone; an increased secretion of antidiuretic hormone retains free water [2, 3].

Ascites occurs as a consequence of these factors, generating a wide range of problems that affect life expectancy starting with general disability, dyspnea, peritoneal infections, renal failure, and finally death [4].

Salt restriction appears as an obvious way to avoid sodium retention and water overload.

It is usually posited that in order to avoid fluid retention, oral sodium intake must be significantly reduced in order to achieve a negative sodium balance. Salt restriction would further decrease fluid accumulation by lowering portal pressure through vascular volume depletion.

All major guidelines in this topic recommend salt restriction in these patients.

However, some authors warn that patients with cirrhotic ascites have low blood sodium levels; therefore, the use of diuretics and sodium restriction could decrease renal perfusion, resulting in further renal impairment and more ascites. Consequently, some randomized controlled studies have suggested there would be no benefit, and even harm, with a sodium-restricted over an unrestricted diet [5, 6].

There are no completed or ongoing systematic reviews on this topic. Therefore, it is critical to provide a high-quality synthesis of current evidence for patients and clinicians about this question.

## Objectives

The aim of this systematic review is to assess the effects of a salt-restricted diet for patients with cirrhotic ascites.

## Methods

A systematic review protocol has been designed and will be reported in line with Preferred Reporting Items for Systematic Reviews and Meta-Analyses Protocols (PRISMA-P) (Additional file 1).

### Criteria for considering studies for this review

#### Types of studies

We will include randomized controlled trials.

#### Types of participants

We will include studies considering adult patients diagnosed with liver cirrhosis of any etiology with confirmed ascites. We will exclude studies of patients with comorbidities that can mimic cirrhotic ascites such as heart and lung insufficiency and patients with previous renal impairment.

#### Types of interventions

- Treatment group: sodium-restricted diet
- Control group: unrestricted

We will also include studies comparing diets with different amounts of sodium, if at least one arm can be considered unrestricted. We will define unrestricted as more than 250 mEq per day.

#### Types of outcomes measures

##### Primary outcomes

- Overall mortality
- Ascites improvement measured by any method

##### Secondary Outcomes

- Sodium plasma levels
- Markers of renal dysfunction
- Hospitalization

## Search methods for identification of studies

We will conduct sensitive electronic searches (with no language or publication restrictions) in EMBASE, MEDLINE, and the Cochrane Central Register of Controlled Trials.

The following strategy will be used to search MEDLINE (PubMed) and adapted for other sources:

- #1 randomized controlled trial [pt]
- #2 controlled clinical trial [pt]
- #3 randomized [tiab]
- #4 placebo [tiab]
- #5 drug therapy [sh]
- #6 randomly [tiab]
- #7 trial [tiab]
- #8 groups [tiab]
- #9 #1 OR #2 OR #3 OR #4 OR #5 OR #6 OR #7 OR #8
- #10 animals [mh] NOT humans [mh]
- #11 #9 NOT #10
- #12 "Sodium, Dietary"[Mesh]
- #13 "Diet, Sodium-Restricted"[MeSH]
- #14 "Sodium Chloride"[Mesh]
- #15 "Sodium"[Mesh]
- #16 salt[ti] OR sodium[ti]
- #17 (restrict* OR low OR reduc* OR intak* OR added OR diet OR consum* OR excess* OR increas* or high) AND (salt OR sodium)
- # 18 #12 OR #13 OR #14 OR #15 OR #16 OR #17
- #19 "Liver Cirrhosis"[Mesh]
- #20 (hepatic or liver) and (fibrosis or cirrho* OR ascit*)
- #21 #19 OR #20
- #21 #11 AND #18 AND #21

Additionally, we will do the following:

- Review clinical practice guidelines and relevant reviews for potentially eligible studies
- Screen the list of references of relevant reviews and included studies identified
- Manually review proceedings of major conferences in the area
- Search for ongoing studies in the World Health Organization (WHO) International Clinical Trials Registry Platform (ICTRP)

## Selection of studies, data extraction, and management

Deduplicated records will be uploaded to Covidence Software (www.covidence.org).

Two reviewers will independently screen the titles and abstracts of all records. The full-text paper of all potentially eligible studies will be obtained for a detailed evaluation and assessed by the same review authors in order to decide on fulfillment of our inclusion criteria.

The same reviewers will extract data using an ad hoc piloted data extraction form. Disagreements will be discussed by all reviewers and judged by an arbiter. The following data will be extracted: (1) general information of the study; (2) study characteristics (study design, sample size, number of arms, methodology of randomization and allocation concealment, blinding, settings); (3) participants (age, gender, ethnicity, method of diagnosis, etiology of cirrhosis, severity of cirrhosis or ascites); (4) interventions and controls (type of interventions, characteristics of salt-restricted diet, duration of treatment or follow-up); (5) outcomes (type of outcome, definition of outcome, time point of assessment); and (6) results.

### Assessment of risk of bias in included studies

Two review authors will independently extract data in pre-designed data extraction and validity assessment forms. A third review author will act as an arbiter in case of disagreement. The methodological quality will be assessed with the Cochrane Collaboration’s tool for assessing risk of bias (Cochrane Handbook (V.5.1.0)), by the following criteria: (1) randomization; (2) allocation concealment; (3) blinding; (4) data integrity; (5) selective reporting; and (6) other bias, such as trial design, baseline similarity of groups, and early cessation of treatment. For all of the studies, the assessment should follow the above six criteria and be categorized as low, unclear, or high risk of bias.

### Measures of treatment effect

Dichotomous data will be determined by using a risk ratio (RR) with 95 % confidence interval (CI), whereas continuous outcomes will be analyzed using mean differences (with 95 % CI) or standardized mean differences (95 % CI) if different measurement scales are used.

### Assessment of heterogeneity

We will assess heterogeneity quantitatively with a formal statistical test (*Q* statistic) and the *I*^2^ statistic. Statistically significant heterogeneity will be defined as at least one positive test (either *P* < 0.10 using the Mantel-Haenszel chi^2^ test or >50 % using the *I*^2^ statistic).

## Data synthesis

If possible, all trials will be combined in a meta-analysis comparing salt-restricted diet versus unrestricted diets. A random effects model will be used for all the analyses. Separate meta-analyses will be presented for specific populations or interventions if statistically significant heterogeneity is explained by some of these or if a convincing subgroup effect is found.

We will perform statistical analysis in accordance with the guidelines for statistical analysis developed by The Cochrane Collaboration, using the statistical software RevMan 5.3.

## Subgroup analysis and investigation of heterogeneity

The following subgroups will be investigated, if possible:

- Ascites severity
- Cirrhosis severity

## Sensitivity analysis

We will perform sensitivity analyses to address the impact of studies with higher or lower bias risk.

## Assessment of reporting bias

We will investigate the publication bias visually with the use of funnel plots. We will base evidence of asymmetry on *P* < 0.10 and present intercepts with 90 % CIs. Outcome reporting bias will be evaluated through discrepancies between the registered protocol and the final publication. We will contact authors for more information if we are not able to find the record of a study in the trials registries.

### Grading the certainty of evidence

We will judge the certainty of the evidence for all outcomes using the Grading of Recommendations Assessment, Development, and Evaluation working group methodology [7].

## Discussion

This systematic review will synthesize scientific evidence for the effect of diet restriction sodium on ascites.

As far as we know, this will be the first systematic review to assess the effectiveness and safety of sodium-restricted diet for ascites in cirrhotic patients. It aims to provide a high-quality synthesis of the current evidence for patients and clinicians about this question.

The main limitation might result from the reduced number and quality of primary studies available. Another limitation might arise from the lack of standardization in the definition of ascites and treatment success or failure. The findings from this systematic review will be valuable in the decision-making process about the effect of sodium-restricted diet as therapy for ascites.

## Additional files

Additional file 1: Corresponds to PRISMA-P checklist. This checklist covers reporting standards for protocol of systematic reviews. The file mentions if these have been covered by the protocol, and where in the text these items are covered.

## Abbreviations

CI: confidence interval
ICTRP: International Clinical Trials Registry Platform
RR: risk ratio
WHO: World Health Organization
