# Supplementary material for: Sodium restriction in patients with cirrhotic ascites: a protocol for a systematic review
Source: Syst Rev. 2016 May 10;5:78. doi: 10.1186/s13643-016-0250-4 (PMC4862086; doi:10.1186/s13643-016-0250-4)
Supplement: Additional file 1: — Corresponds to PRISMA-P checklist. This checklist covers reporting standards for protocol of systematic reviews. The file mentions if these have been covered by the protocol, and where in the text these items are covered. [file 13643_2016_250_MOESM1_ESM.docx]

**PRISMA-P (Preferred Reporting Items for Systematic review and Meta-Analysis Protocols) 2015 checklist: recommended items to address in a systematic review protocol***

| Section and topic | Item No | Checklist item |
| --- | --- | --- |
| ADMINISTRATIVE INFORMATION  **Title: Sodium restriction in patients with cirrhotic ascites: protocol for a systematic review** | | |
|  |  |  |
| Identification | 1a | Identify the report as a protocol of a systematic review: **YES. Identified as protocol in the title.** |
| Update | 1b | If the protocol is for an update of a previous systematic review, identify as such. **This is the first document and not an update** |
| Registration | 2 | If registered, provide the name of the registry (such as PROSPERO) and registration number. **PROSPERO CRD42015022161** |
| Authors: |  |  |
| Contact | 3a | Provide name, institutional affiliation, e-mail address of all protocol authors; provide physical mailing address of corresponding author. **YES. Names, affiliations and emails of all authors provided. Physical mailing address of corresponding author provided.** |
| Contributions | 3b | Describe contributions of protocol authors and identify the guarantor of the review. **YES. Contributions and guarantor described in page 10** |
| Amendments | 4 | If the protocol represents an amendment of a previously completed or published protocol, identify as such and list changes; otherwise, state plan for documenting important protocol amendments. **This is the original protocol. Important protocol amendments will be documented and submitted to the registering body in compliance with PROSPERO registration** |
| Support: |  |  |
| Sources | 5a | Indicate sources of financial or other support for the review. **Yes. Described in page 10** |
| Sponsor | 5b | Provide name for the review funder and/or sponsor. **Yes. Described in page 10** |
| Role of sponsor or funder | 5c | Describe roles of funder(s), sponsor(s), and/or institution(s), if any, in developing the protocol. **Yes. Described in page 10** |
| INTRODUCTION | | |
| Rationale | 6 | Describe the rationale for the review in the context of what is already known. **Yes. Described in pages 2 and 3.** |
| Objectives | 7 | Provide an explicit statement of the question(s) the review will address with reference to participants, interventions, comparators, and outcomes (PICO). **Yes. Described in page 5.** |
| METHODS | | |
| Eligibility criteria | 8 | Specify the study characteristics (such as PICO, study design, setting, time frame) and report characteristics (such as years considered, language, publication status) to be used as criteria for eligibility for the review. **Yes. Described under ‘Methods/Criteria for considering studies for this review’ in page 5.** |
| Information sources | 9 | Describe all intended information sources (such as electronic databases, contact with study authors, trial registers or other grey literature sources) with planned dates of coverage. **Yes. Described under ‘Methods/Search methods for identification of studies’ in page 6.** |
| Search strategy | 10 | Present draft of search strategy to be used for at least one electronic database, including planned limits, such that it could be repeated. **Yes. Described under ‘Methods/Search methods for identification of studies’ in pages 6 and 7.** |
| Study records: |  |  |
| Data management | 11a | Describe the mechanism(s) that will be used to manage records and data throughout the review. **Yes. Described in page 7.** |
| Selection process | 11b | State the process that will be used for selecting studies (such as two independent reviewers) through each phase of the review (that is, screening, eligibility and inclusion in meta-analysis). **Yes. Described in page 7.** |
| Data collection process | 11c | Describe planned method of extracting data from reports (such as piloting forms, done independently, in duplicate), any processes for obtaining and confirming data from investigators. **Yes. Described in page 7.** |
| Data items | 12 | List and define all variables for which data will be sought (such as PICO items, funding sources), any pre-planned data assumptions and simplifications. **Yes. Described in page 5.** |
| Outcomes and prioritization | 13 | List and define all outcomes for which data will be sought, including prioritization of main and additional outcomes, with rationale. **Yes. Described under ‘Methods/Criteria for considering studies for this review’ in page 5 and 6.** |
| Risk of bias in individual studies | 14 | Describe anticipated methods for assessing risk of bias of individual studies, including whether this will be done at the outcome or study level, or both; state how this information will be used in data synthesis. **Yes. Described under ‘Methods/** **Risk of bias in included studies’ in page 8.** |
| Data synthesis | 15a | Describe criteria under which study data will be quantitatively synthesised. **Yes. Described under ‘Methods/Data synthesis ’ in pages 8 and 9.** |
|  | 15b | If data are appropriate for quantitative synthesis, describe planned summary measures, methods of handling data and methods of combining data from studies, including any planned exploration of consistency (such as I^2^, Kendall’s τ). **Yes. Described in pages 8 and 9.** |
|  | 15c | Describe any proposed additional analyses (such as sensitivity or subgroup analyses, meta-regression). . **Yes. Described under ‘Methods/Subgroup analysis and investigation of heterogeneity’ and under ‘Methods/sensitivity analysis’ in pages 9.** |
|  | 15d | If quantitative synthesis is not appropriate, describe the type of summary planned. **Yes. Described under ‘Methods/Data synthesis ’ in page 8 and 9 .** |
| Meta-bias(es) | 16 | Specify any planned assessment of meta-bias(es) (such as publication bias across studies, selective reporting within studies). **Yes. Described under ‘Methods/Assessment of reporting biases’ on page 9** |
| Confidence in cumulative evidence | 17 | Describe how the strength of the body of evidence will be assessed (such as GRADE). **Yes. Described under ‘Methods/** **Grading the quality of evidence’ in page 9** |

*From: Shamseer L, Moher D, Clarke M, Ghersi D, Liberati A, Petticrew M, Shekelle P, Stewart L, PRISMA-P Group. Preferred reporting items for systematic review and meta-analysis protocols (PRISMA-P) 2015: elaboration and explanation. BMJ. 2015 Jan 2;349(jan02 1):g7647.*
